# Outcomes of Transplantation of Single Kidneys From Pediatric Donors Into Adult Recipients

**DOI:** 10.7759/cureus.52399

**Published:** 2024-01-16

**Authors:** Ashraf Reyad, Nikhil A Reddy, Debra Meeks, James Pittman, Noah Zanville, Anna Curtis, Machaiah Madhrira, Sridhar R Allam

**Affiliations:** 1 Transplant Institute, Medical City Fort Worth, Fort Worth, USA; 2 Surgery, Burnett School of Medicine at TCU (Texas Christian University), Fort Worth, USA; 3 North Texas Division, HCA Healthcare Research Institute, Fort Worth, USA; 4 Clinical Operations Group, HCA Healthcare, Nashville, USA; 5 Clinical Services Group, HCA Healthcare, Nashville, USA; 6 Transplant Nephrology, PPG Health, Fort Worth, USA; 7 Internal Medicine, Burnett School of Medicine at TCU (Texas Christian University), Fort Worth, USA

**Keywords:** hyperfiltration injury, en bloc kidneys, renal transplant outcomes, small pediatric donors, single kidney transplantation

## Abstract

Background

Organs from extreme ages have been sought after to help increase the donor pool and alleviate transplantation wait times. There has been a growing evolution of the use of pediatric donor kidneys, including the use of en bloc kidneys (EBK), to now separating them into single kidneys (SKT), allowing for transplantation of two recipients. This study reports our outcomes utilizing SKT.

Methods

A retrospective review of all SKT performed from 2014 to 2022 at our center was conducted. Donors >8 years of age or >25 kg in weight were excluded. Donor and recipient characteristics and outcomes were analyzed, comparing <18 kg and ≥18 kg donor cohorts.

Results

Between 2014 and 2022, 81 adults received SKT. Recipients’ mean age, weight, and body mass index were 49 years (22-74), 74 kg (39-136), and 26.4 mg/m^2^ (19.6- 39.8), respectively. Donors’ mean age, weight, and kidney size were 35.7 months (8-96), 17.8 kg (8-25), and 7.2 cm (4.5-8.5), respectively. At one year post-transplant, patient survival was 100%, graft survival was 98.7%, mean serum creatinine was 1.25 mg/dL, and mean glomerular filtration rate (GFR) was 68.3 ml/min. Hyperfiltration injury was seen in 43.75% of recipients. None of the outcomes correlated with any of the donor or recipient characteristics.

Conclusion

Our study shows excellent short-term outcomes of single pediatric kidney transplantation in adult recipients. Exploring a lower donor weight cut-off for SKT, compared to the current Organ Procurement and Transplantation Network's (OPTN's) ≥18 kg, could expand the organ pool and lead to an increased number of transplants.

## Introduction

Organs from extreme ages have been sought after to help increase the donor pool and alleviate transplantation wait times. Among these, pediatric donor kidneys have been an important source of organs since the 1970s [1]. There has been a growing evolution in the use of pediatric donor kidneys, including the use of en bloc kidneys (EBK), to now separating them into single kidneys (SKT), allowing for the transplantation of two recipients. Splitting EBK to SKT can depend on certain donor criteria such as age, weight, and kidney size. 

While an important step for the field, concerns regarding the transplantation of pediatric kidneys include a higher incidence of technical complications (vascular or urinary), increased incidence of cellular rejection, higher rate of delayed graft function, and size mismatches with potentially insufficient nephron mass or hyper-filtration injury risk [2-4]. In an era where transplant center outcomes are increasingly scrutinized, there is significant hesitation about the utilization of kidneys from small pediatric donors for fear of suboptimal outcomes.

More recent data have shown similar graft survival with EBK compared to standard adult living donor and deceased donor kidney transplants [5]. However, by splitting the en bloc kidneys, the number of recipients potentially receiving a transplant can double. The balance between optimizing transplant outcomes and maximizing the number of transplants by safely splitting EBK is driven by a triad of donor criteria, recipient acceptance criteria, and the transplant surgeon’s technical skill.

In September 2019, the Organ Procurement and Transplantation Network (OPTN) implemented a new policy to allocate kidneys from deceased donors <18 kg as en bloc by host Organ Procurement Organizations (OPO) [6]. Although these kidneys are allocated in the same sequence as donors with a Kidney Donor Profile Index (KDPI) of 0-20% via an en-bloc specific match run, the current KDPI lacks an en-bloc coefficient that is reflective of the relative risk of the graft failure from the en-bloc transplantation. In 2022, OPTN released the report of two-year trends of the allocation of pediatric single and en bloc kidneys post-implementation of this policy [7]. The percentage of en-bloc kidney transplants nationally, accounting for 1.03-1.11% of all deceased donor kidney transplants, has not significantly changed. However, the number of single kidney transplants from donors <18 kg significantly decreased from 214 (19.2%) pre-policy era to 114 (11.5%) post-policy era, while slightly increasing the en bloc transplants from 335 (56.5%) pre-policy era to 353 (64.3%) post-policy and maintaining a non-utilization rate of 23.6% and 23.9%, respectively. On the other hand, for donors 18-25 kg, there was a decrease in en bloc use from 24 to 20 cases and a slight increase in single kidneys from 214 to 217, pre and post-policy, respectively. Only 38% of transplant centers performed at least one en bloc transplant during the reporting period. These data reflect an opportunity for improvement in the utilization of single pediatric kidneys from donors under 18 kg.

Herein, we present the results from our institution’s experience of the utilization of single pediatric kidneys for transplantation in adult recipients. While prior data reporting outcomes using the Scientific Registry of Transplant Recipients (SRTR) registry, a single-center experience provides better granularity of data and consistent technical expertise.

## Materials and methods

We performed a single-center, retrospective, cross-sectional review of recipients who received a single kidney transplantation from pediatric donors at our institution between 2014 and 2022. This study was approved by the institutional review board responsible for overseeing human subjects research at our institution (MCOR-01). Informed consent from patients was not obtained, as this was a retrospective study using deidentified data. Data collection included donor and recipient characteristics, operative data, and recipient outcomes. Electronic medical records and paper charts were reviewed.

Eligibility criteria for recipients at our center to receive SKTs include recipient weight <100 kg, absence of hyper-coagulable risk factors, or history of primary focal segmental glomerulosclerosis (FSGS). SKTs from pediatric donors >8 years of age or >25 kg of weight were excluded from the study. Recipient to donor weight ratio (RW: DW) is typically <10. We generally have not split EBK if they were <5 cm or donors were <5 kg, however, we made rare exceptions for recipients who were < 65 kg and had very small bladders that only a single ureteral anastomosis was feasible. SKT is performed in a similar technique to adult kidney back-tabling. As this study period spans both pre and post-OPTN policy eras, there has been a variation in how the kidneys were offered, en bloc vs split. Generally, all donors ≥18 kg have been offered as single. Donors >5 kg - <18 kg are offered as en-bloc. The decision to split is based on the availability of suitable recipients.

Some perioperative considerations for SKT included the administration of 3000 units of IV heparin at least three minutes prior to clamping. Heparin drip was started immediately after reperfusion at a rate of 300 units/hour. A 4.7 Fr pediatric double J stent is used for the neoureterocystostomy anastomosis. We routinely placed perinephric JP drains to decrease any mass effects from potential postoperative collections and to test for urine leaks. Prior to skin closure, a transplant renal ultrasound (US) was performed to confirm the kidney orientation had not resulted in vessel kinking or twisting.

Recipient blood pressure was closely managed to avoid systolic blood pressure (SBP) >140 postoperatively. The heparin drip was titrated to the activated partial thromboplastin time (aPTT) goal of 40-50 seconds. We had a low threshold for repeated imaging if clinically indicated postoperatively. Patients were discharged on aspirin 325 mg by mouth daily for at least three months postoperatively.

Immunosuppression

Rabbit anti-thymocyte globulin was generally used for induction, cumulatively dosed at 4.5 mg/kg for low immunological risk recipients or 6 mg/kg for high immunologic risk. Steroid induction was initiated at 500 mg and tapered to 20 mg daily over four days. The maintenance steroid was reduced to 5 mg daily over 6-12 weeks. Tacrolimus was started at a conservative dose and slowly titrated up to a goal of 8-10 ng/mL, keeping in mind any potential vasoconstrictive effects that tacrolimus may have on pediatric vessels. Recipients also received mycophenolate sodium 720 mg twice daily.

Statistical analyses

Statistical analyses were performed using SAS version 9.4 (SAS Institute, Cary, NC, United States). Descriptive statistics of continuous variables are presented as means or medians with ranges. Categorical variables are presented as proportions of the total sample. Two-tailed χ2 or Fisher's exact tests were used to compare categorical variables; Mann-Whitney U tests were used to compare continuous variables between groups of patients who experienced complications, including a hyperfiltration injury or suboptimal glomerular filtration rate (GFR) levels. Unless otherwise stated, an α level of 0.05 was used to determine significance.

## Results

Between 2014 and 2022, we performed a total of 802 kidney transplants. We performed 81 SKT and 35 EBK in this time period, equating to almost 15% of our total transplants.

Recipient demographics

Recipient demographics are summarized in Table 1. Recipients’ mean age was 49 years (range: 22-74). Fifty-three percent (53%) were male; 31% were Caucasian, 23.5% were African American, 23.5% were Hispanic, 16% were Asian, and 6% were multiracial. Seventy-six point five percent (76.5%) had a calculated panel reactive antibody (cPRA) score of zero while only 6.2% had a high cPRA (> 80%). The recipients’ mean weight was 74 kg (range: 39 - 126 kg) and the mean body mass index (BMI) was 26.4 (range: 19.6-39.8).

**Table 1:** Recipient demographics

Recipient Characteristic	n=81
Mean Age, Years (range)	49 (22-74)
Sex (%)	
Male	53%
Female	47%
Racial Ethnicity (%)	
White	31%
Black	23.50%
Hispanic	23.50%
Asian	16%
Multiracial	6%
Mean Calculated Panel Reactive Antibody, % (range)	10 (0-99.4)
Mean Weight, kg (range)	74 (39-126)
Mean Body Mass Index, kg/m2 (range)	26.4 (19.6-39.8)

Donor demographics 

Donor demographics are summarized in Table 2. Ninety-one percent (91%) of donors had brain death at the time of donation. The mean cold ischemic time was 14.9 hours (range: 4.5-36 hours). The mean donor age was 35.7 months (range: 8-96 months) with a mean kidney donor profile index (KDPI) of 63% (range: 42%-89%). The mean donor terminal creatinine was 0.48 mg/dL (range: 0.1-1.6). The mean donor weight was 17.8 kg (range: 8-25). Eight SKT were from donors 8-10 kg, 32 were >10-15 kg, 24 were >15-20 kg, and 17 were >20-25 kg. The mean donor kidney length was 7.2 cm (range: 4.5-8.5). One was 4.5 cm, two were 5 cm, seven were 6 cm, seven were 6.5 cm, 26 were 7 cm, 13 were 7.5 cm, and 25 were >7.5 cm.

**Table 2:** Donor demographics

Donor Characteristic	n=81
Brain dead donor (%)	91%
Mean cold ischemic time, hours (range)	14.9 (4.5-36)
Mean age, months (range)	35.7 (8-96)
Mean kidney donor profile index % (range)	63% (42-89)
Mean donor terminal creatinine, mg/dL, (range)	0.48 (0.1-1.6)
Mean donor weight, kg (range)	17.8 (8-25)
Distribution of donor weight range (n, %):	
8 kg -≤ 10 kg	8 (9.9%)
10 kg -< 15 kg	32 (40%)
≥15 kg -< 18 kg	16 (19.8%)
≥18 kg -≤ 25 kg	25 (30.8%)
Mean donor kidney length, cm (range)	7.2 (4.5-8.5)
Distribution of kidney size (n, %)	
4.5 cm	1 (1.2%)
5 cm	2 (2.4%)
6 cm	7 (8.4%)
6.5 cm	7 (8.4%)
7 cm	26 (31.2%)
7.5 cm	13 (15.6%)
≥7.5 cm	25 (30%)

Outcomes

For outcomes, keeping up with the new OPTN policy change, we grouped our cohorts into recipients of kidneys from donors <18 kg (n=56) vs. donors ≥18 kg (n=25). We explored a calculation that would incorporate factors like donor weight, recipient weight, and kidney size by using a calculation we named Renal Adjusted Weights and Size (RAWS) score. The calculation was performed retrospectively to see if it can be used as a safe guide for the feasibility of splitting pediatric en bloc kidneys. RAWS was obtained by an equation of recipient weight (kg) divided by the multiple of donor weight (kg) and donor kidney size (cm).

We did see statistically significant differences between the two cohorts for donor weight, kidney size, donor age, mean RAWS, and Rw: Dw (Table 3). This was expected as donors <18 kg are expected to be smaller in size, of a younger age, and therefore matched with proportionally smaller recipients. Of note, all recipients in our study had an RAWS score <1.6.

**Table 3:** Donor and recipient characteristics and outcomes after transplantation of single kidneys from pediatric donors under 18 kg vs. ≥18-25 kg RAWS, recipient weight (kg)/(donor weight (kg) x kidney size (cm); RW: DW, recipient to donor weight ratio; GFR, glomerular filtration rate; UPCR, urine protein creatinine ratio. Two-tailed χ2 or Fisher's exact test was used to compare categorical variables with a p-value <0.05 was considered statistically significant.

Metric	Donors <18 kg (n=56, functional grafts 55)	Donors ≥18-25 kg (n=25)	P-value
Patient 1-year survival (%)	100%	100%	1
Graft 1-year survival (%)	98.20%	100%	0.5
Delayed graft function (%)	5.45% (3/55)	4% (1/25)	0.78
Mean creatinine at 1 year, mg/dL (range)	1.21 (0.5-3.4)	1.09 (0.6-1.7)	0.3
Mean RAWS (range)	0.83 (0.3-1.57)	0.65 (0.33-1.01)	<0.0001
RW: DW	5.8 (±1.46)	3.71±0.84	<0.0001
GFR, ml/min mean, (SD)	70.05 (±23.13)	63.84 (±23.31)	0.33
Donor weight mean (kg, SD)	12.8±2.5	20.9±2.34	<0.0001
Kidney size mean (cm, SD)	7.12±0.87	7.62±0.96	0.02
Donor age mean (month, SD)	29.79±17.97	48.92±16.1	0.00002
Recipient weight mean (kg, SD)	71.57±1.46	76.92±16.8	0.13
Mean UPCR (range)	0.52 (0.01-2.8)	0.49 (0.15-2.8)	0.53

Patient survival

At one year post-transplant, recipient survival was 100.0%, with no statistical significance between our two cohorts. This compares well with the OPTN two-year report, which showed 94.9% one-year patient survival for SKT. Two patient deaths with functioning grafts occurred one-year post-transplant during the coronavirus disease 2019 (COVID-19) pandemic’s first and second waves, respectively. One of these patients had pre-existing pulmonary fibrosis and succumbed to COVID-19 pulmonary sequelae at 28 months post-transplant, with normal kidney function at the time of death. The second patient had a serum creatinine of 1.8 mg/dL at the time of hospital admission for COVID-19 but developed acute kidney injury along with multiorgan failure and died at 30 months post-transplant.

Graft survival

At one year post-transplant, graft survival was 98.2% for donors <18 kg vs 100% for donors ≥18 kg. This was not significant statistically. The only graft loss was due to vascular dissection requiring allograft explantation on postoperative day one. This kidney was from a 10.9 kg donor. The graft survival in our study was higher than OPTN-reported EBK and SKT one-year graft survival of 93.1% and 92.6%, respectively. It is notable that four patients developed thrombotic microangiopathy (TMA) immediately post-transplant. Three of these four patients were diagnosed with atypical hemolytic syndrome (aHUS), and one patient was diagnosed with thrombotic thrombocytopenic purpura (TTP). Three of the four were in the <18 kg cohort group. All of these patients’ grafts were salvaged with expedited recognition and appropriate treatment during their index transplant hospitalization.

Delayed graft function (DGF)

The DGF rate was 5.45% in the <18 kg donor group vs 4% in the ≥18 kg donor group and was not statistically significant. One of these patients was diagnosed with TMA. The immediate recognition of TMA in this recipient allowed for early diagnosis and treatment of TMA in the mate kidney recipient at our center. For patients with DGF, we performed daily renal transplant ultrasounds (US) during their index transplant hospitalization while keeping a low threshold to repeat if a decrease in urine output was noted. We also had a low threshold to return to the operating room for any technical concerns.

Graft function

The mean recipient serum creatinine and GFR at one-year post-transplant were 1.21 mg/dL and 70.05 ml/min in the <18 kg cohort vs 1.09 mg/dL and 63.8 ml/min in the ≥18 kg cohort. They were not statistically significant. The mean urine protein creatinine ratio (UPCR) was 0.52 in the <18 kg cohort vs 0.49 in the ≥18 kg cohort, and these were not statistically significant. Hyperfiltration injury was seen in 48.2% in the <18 kg cohort vs 32% in the ≥18 kg cohort. These patients were treated with angiotensin-converting enzyme inhibitors (ACEi) or angiotensin receptor blockers (ARB). The mean time to the development of proteinuria was 157 days post-transplant (range: 41-36). Prompt improvement of UPCR was seen with the initiation of ACEi or ARB. None of the patients that developed proteinuria and were managed with ACEi or ARB had deterioration of allograft function.

We investigated donor and recipient risk factors for GFR <45 ml/min, <30 ml/min, and hyperfiltration injury for both the <18 kg and ≥18 kg cohorts and found no significant correlation of these outcomes with donor age, donor weight, kidney size, recipient weight, or recipient to donor weight ratio (Table 4).

**Table 4:** Correlation of suboptimal GFR and hyperfiltration injury with donor and recipient characteristics after transplantation of single kidneys from pediatric donors under 18 kg vs. ≥18-25 kg GFR, glomerular filtration rate; RW: DW, recipient-to-donor weight ratio; UPCR, urine protein creatinine ratio Mann-Whitney U tests were used to compare continuous variables between groups with a p-value of <0.05 was considered statistically significant.

Metric	Donors <18 kg (n=56, functional grafts 55)	P-value	Donors ≥18-25 kg (n=25)	P-value
GFR <30 (n, %)	3 (5.45%)		2 (8%)	
Characteristic				
Donor weight (kg, SD)	13±1.89	1	23.35±2.33	0.16
Kidney size (cm, SD)	7.50±0.5	0.45	7.75±1.06	0.8
Donor age (months, SD)	35.67±10.97	0.27	66.00±8.48	0.13
Recipient weight (kg, SD)	73.8±22.98	0.96	81.00±15.56	0.51
RW: DW (ratio, SD)	5.77±1.93	0.87	3.45±0.32	0.51
GFR <45 (n, %)	5 (9.09%)		3 (12%)	
Characteristic				
Donor weight (kg, SD)	13.44±1.60	0.71	21.57±3.50	0.77
Kidney size (cm, SD)	7.80±0.57	0.05	7.50±0.87	0.93
Donor age (months, SD)	35.40±8.26	0.12	60.00±12.00	0.27
Recipient weight (kg, SD)	75.2±18.82	0.8	83.83±12.05	0.22
RW: DW (ratio, SD)	5.65±1.43	0.99	3.96±0.91	0.77
Hyperfiltration injury (UPCR >0.3) %	27 (48.2%)		8 (32%)	
Characteristic				
Donor weight (kg, SD)	13±2.2	0.73	20.51±2.85	0.4
Kidney size (cm, SD)	7.05±0.84	0.56	7.63±1.13	0.88
Donor age (months, SD)	31.48±17.62	0.35	39.88±18.29	0.06
Recipient weight (kg, SD)	69.57±14.59	0.11	74.13±10.26	0.38
RW: DW (ratio, SD)	5.43±1.23	0.11	3.70±0.85	1

Postoperative return to the operating room

Six (7.4%) recipients had to return to the operating room. One patient was noted to have a thrombosed graft requiring explantation on postop day one. Four patients required an evacuation of hematoma due to compressive changes with mass effect on ultrasound. One patient had a sudden decrease in urine output and perfusion on the US. This revealed 30-40% thrombosis of the kidney, and intraoperative kidney biopsy revealed TMA.

Acute rejection

Two (2.5%) patients had acute rejection in their first year. Both of these patients had a cPRA of 0%. One was an African American patient who developed Banff 1b acute cellular rejection (ACR) at five months post-transplant. The second patient was a re-transplant who developed Banff 2a ACR about two months post-transplant. Both patients were in the <18 kg group and had successful treatment of their rejection with normal allograft function thereafter.

Ureteral complications

One (1.5%) patient developed ureteral stricture and required nephroureteral stent placement by interventional radiology. None of the patients had urine leak complications.

## Discussion

This single-center, retrospective study demonstrates excellent one-year graft survival (98.7%) and patient survival (100%) of SKT from pediatric donors. Although there is no real dispute when considering splitting pediatric EBKs from donors >18 kg, to this date, the decision to split kidneys from en bloc donors below 18 kg is controversial and unchallenged. Furthermore, the utilization of these kidneys decreased after the 2019 OPTN allocation policy change [6,7].

Despite the studies showing en bloc transplantation as similar graft survival compared to standard adult living and deceased donor transplants [5,8]. Suneja et al. reported that up to 10% of kidneys from donors ≤ 20 kg are discarded [9] and per the OPTN post-policy change two-year report, the discard rate worsened from 15% to 18% rate for donors <18 kg [7]. Technical concerns associated with this procedure remain a barrier, including up to 10% vascular thrombosis rate and ureteral complications. Donor body weight in en bloc kidney transplantation was investigated by Peng et al. (285 EBK from 2015-2019) [10]. Donor weight thresholds of <5 kg versus 5- <20 kg vs >20 kg were found to have survival rates of 71.4% vs 89.5% vs. 97.3%, respectively (p < 0.05) Similarly, Sureshkumar et al. analyzed SRTR data and concluded graft failure rate of single pediatric kidney transplants was consistently lower when donor weight exceeded 10 kg [11]. Several other studies also reported comparable patient and graft survival from utilization of kidneys from donors ≤ 5 yrs as SKT or EBK [12,13]. The only graft loss in our study occurred from a 10.9 kg, 26-month-old brain-dead donor that had a terminal creatinine of 0.36 mg/dL. The vascular thrombosis rate in our study was 1.3%, which is comparable to the adult graft vascular thrombosis rate. In fact, 8/81 (9.8%) of donors in our study were <10 kg with 100% one-year graft and patient survival in that subgroup. Our one-year patient and graft survival in both <18 and ≥18 kg were excellent, not significantly different, and exceeded the two-year OPTN reports. In our <18 kg cohort, the graft survival was higher than the OPTN en bloc graft survival for that weight group. This again calls for serious evaluation of splitting en bloc kidneys into single kidneys for transplantation into appropriate candidates and that donor weight should not be the only consideration factor.

Maluf et al. reviewed the OPTN database from January 2005 and 2010 data on utilization patterns for kidneys ≤20 kg [14]. They showed that the frequency with which at least one kidney was transplanted increased steeply with donor weight; in fact, it was at the 13 kg donor weight when the rate of SKT (52%) transplants exceeded EBK (48%). In our cohort, 69.1% were <18 kg and the largest cohort was in the ≥10-<15 kg donor group. Maluf et al. also showed that only donor weight was significantly associated with graft failure, while donor age and recipient BMI showed a trend. Furthermore, the 11 kg donor group had 81.4% 1-year graft survival comparable to the 81.7% survival for the adult ECD group and 86.3% survival for 20 kg donors. Our center previously reported our early experience with SKT in 2018 [15]. As our volume and comfort with the utilization of kidneys from smaller pediatric donors evolved, our practice now primarily considers splitting en bloc kidneys into single kidneys for transplanting two recipients. Chen et al. compared SKT and EBK from donors ≤10 kg and showed that the 1-year and 3-year death-censored graft survival for SKT was not significantly different than EBK (95% vs. 80%, p = 0.38) [16]. Although we agree donor weight is critical, in our experience, technical comfortability and recipient selection are equally important for optimal outcomes. Supporting this statement, the study by Maluf et al. demonstrated that for centers utilizing kidneys from small pediatric donors, center experience (defined as >5 transplants/year) was associated with estimated outcome differentials of 40% for SKT vs 30% for EBK, with no outcome differences observed at the upper end of the weight spectrum [14]. Also, the OPTN two-year post-policy report found that only 38% of transplant centers performed at least one transplant from pediatric donors. There is substantial literature that center/surgeon volume and expertise are critical outcome determinants for technical surgical procedures [17-19]. Our center performed >10 SKT/year, and our graft survival rates were not significantly different between the <18 kg and ≥18 kg donor cohorts. Maluf et al.'s study estimated modest gains by splitting en bloc kidneys from the 8-9 kg donors with a higher risk of graft loss; however, splitting en bloc kidneys from donors >10 kg and >12 kg would lead to 300 and 225 additional transplants respectively [14].

Proteinuria likely due to hyperfiltration injury from size mismatch between SKT and adult recipients was noted in 48.2% and 32% of patients in <18 kg and ≥18 kg cohorts, respectively. These rates were not significantly different. We also found no significant correlation between hyperfiltration injury and donor weight, kidney size, donor age, or recipient weight. Nonetheless, these patients were all treated with ACEi or ARB, with improvement in proteinuria. Hyperfiltration injury did not impact their one-year graft survival. We transplanted eight kidneys from donors <10 kg. These patients had a mean Rw: Dw ratio of 7.8 (range 6.4-10), RAWS score of 1.2 (range 0.9-1.57), and donor mean serum creatinine of 1.02 (range 0.6-1.37). Hyperfiltration injury in this subgroup was noted to be 37.5% (3 out of 8), which was not higher compared to the overall study group. Borboroglu et al. reported that SKT recipients did not experience hyperfiltration injury if the donor's weight exceeded 14 kg and the kidney length exceeded 6 cm [20]. Felix et al. reported that, in their study of 47 SKT from donors <5 years of age and a median follow-up of 2.3 years, 55% of recipients had histological signs of early hyperfiltration injury that improved to a rate similar to standard criteria donors and higher median GFR at the end of follow up (80 vs 55 ml/min/1.73 m^2^) [21].

We generally selected recipients <90 kg in weight for SKT. However, we transplanted eight patients that weighed ≥90 kg. In this subgroup, the mean recipient weight was 99.3 kg (range: 90-126 kg). The mean recipient BMI for this subgroup was 32 (range: 25-39.9). The donor ages were generally ≥36 months and had a mean weight of 18.5 kg (range: 13.7-25 kg). The mean Rw: Dw was 5.6 (range: 3.68-7.12) and the mean RAWS score was 0.71 (range: 0.53-1.02). The recipients’ mean serum creatinine at one year was 1.63 mg/dL (range: 1.0-2.5). Three of the eight (37.5%) recipients developed hyperfiltration injuries with no significant correlation to the Rw: Dw or RAWS score.

Four of the 80 (5%) recipients were diagnosed with TMA post-transplant. This is higher than the rates of 1-2% reported in adult kidney transplant recipients. The combination of smaller vasculature, tedious, longer back-tabling, and early use of tacrolimus could be possible risk factors. It is critical to monitor these recipients closely for early recognition of TMA, to allow for prompt initiation of treatment. Additionally, the DGF rate for SKT from pediatric donors in our study was low, at 5% (4 of 80), but DGF may be an early indicator of pathological processes like TMA and should alert a high index of suspicion.

The overall reoperation rate in the first 30 days post-transplant was high at 7.4% (6 out of 81). Four of these were for the evacuation of a hematoma. All hematoma washouts occurred within two days postop and did not have a long-term effect on graft survival. This could be explained by the difficulty in identifying and performing ideal meticulous small vessel ligations with these pediatric kidneys. One re-exploration was due to changes in US perfusion and urine output revealing 30-40% graft thrombosis. This allowed for intraoperative kidney biopsy and diagnosis of TMA.

Strengths and limitations

To our knowledge, this is the largest single-center study to describe the experience of transplanting single kidneys from pediatric donors into adult recipients. However, our study has some limitations. The main limitations were the retrospective nature of the study, a small cohort size, and a short-term follow-up. There is obvious selection bias among the donors and recipients, which is intentional to match these pediatric kidneys to appropriate adult recipients to maximize the number of transplants and to achieve optimal outcomes. Subgroups of donor kidneys <10 kg and recipients >90 kg were smaller. Finally, our study does not identify a lower threshold or safe cutoff for donor or recipient characteristics outside of our center criteria. However, our study does challenge the current OPTN policy of allocating kidneys from donors <18 kg as en bloc, which has resulted in decreased numbers of SKT and lost opportunities for transplanting more recipients.

## Conclusions

Transplantation of single kidneys from small pediatric donors into appropriate adult recipients is safe and effective and can increase the number of transplants performed. Using thresholds such as Rw: Dw ≤10 and RAWS <1.6 can serve as a safe guide for recipient selection, but further investigation into a lower limit or cut-off threshold is warranted in the future. In addition, these formulas could not predict hyperfiltration injury risk, which is frequently seen with these kidneys. This risk of hyperfiltration injury suggests excluding recipients who are at high risk of developing proteinuria post-transplant such as those with primary FSGS. The risk of TMA is also higher with these kidneys. Therefore, close monitoring with early recognition and prompt management are of utmost importance in this patient population.
